# Acute Stress Induced Catatonic Psychosis in an Adolescent: A Case Report

**DOI:** 10.1192/j.eurpsy.2024.1515

**Published:** 2024-08-27

**Authors:** E. Yerlikaya Oral

**Affiliations:** Department of Child and Adolescent Psychiatry, University of Health Sciences, Bakirkoy Prof. Dr. Mazhar Osman Research and Training Hospital for Psychiatry, Neurology and Neurosurgery, Istanbul, Türkiye

## Abstract

**Introduction:**

Childhood maltreatment(CM) can precipitate a range of psychiatric disorders in individuals. Some research show that CM rates are as high as 85% in schizophrenia spectrum disorders (Larsson *et al.* 2013). This case report explores an instance of acute catatonic psychosis in an adolescent following a significant episode of physical and emotional abuse.

**Objectives:**

The aim is to elucidate the clinical presentation, diagnosis, and treatment of trauma-induced acute catatonic psychosis in an adolescent. The report seeks to emphasize the potential link between acute trauma and severe psychiatric disorders in young individuals.

**Methods:**

A thorough review of the patient’s clinical records was undertaken, focusing on psychiatric history, symptoms, treatment trials and responses. In parallel, an extensive literature review was conducted to understand the current knowledge on the association between acute traumatic stress and acute psychosis with catatonia.

**Results:**

The patient, a 16-year-old female, presented with severe symptoms of catatonia and psychosis including mutism, posturing, stupor, negativism, auditory hallucinations and persecutory delusions, in addition; eating refusal, urinary and fecal incontinence. Symptoms started immediately following physical and emotional abuse that occurred 10 days ago. She was hitted, insulted and detained for 2 days by her parent’s friends. Abuse reported to social services and judicial authorities. All laboratory and neurologic examinations performed to exclude an organic pathology. No pathologic results founded. Olanzapine 5 mg/day and lorazepam 0.5 mg/day started and titrated to 30 mg/day and 3.75 mg/day doses. Biperiden 4 mg/day started due to extrapyramidal side effects. A significant improvement observed about her catatonic and positive psychotic symptoms but she still had acute stress disorder symptoms. Trauma-focused cognitive-behavioral therapy added to her treatment. Family-based interventions examined for CM. She discharged in full remission after eight weeks of hospital stay. Lorazepam dose reduced and stopped before discharge.

**Conclusions:**

Neurobiological models are trying to enlight the association between experiencing highly stressful or traumatic events, such as child abuse, may impact on later expression of psychotic disorders by increasing stress sensitivity to later adversity (Fares-Otero *et al.* 2023). This case underscores the potential of acute traumatic stress to precipitate severe psychiatric disorders, including catatonia. It highlights the importance of comprehensive clinical evaluations and the inclusion of trauma history in children presenting with acute psychiatric symptoms. The findings advocate for the integration of trauma-focused interventions in the treatment of similar cases. Further research is needed to understand the pathophysiological mechanisms underlying this association and to develop effective treatment strategies for this vulnerable population.

**Disclosure of Interest:**

None Declared

